# 

**Published:** 1894-08

**Authors:** 


### INDEX TO VOLUME XI., NEW SERIES.

[OLD SERIES VOL. XXVI.]

CLINICAL LECTURES—

Chronic Bronchitis. Endocarditis .......... 641
Chronic Gastritis. Pernicious Anemia. Distention of Stomach. (Delafield.) .......... 513

ORIGINAL COMMUNICATIONS—

Abortion and Infanticide. (Eve.) .......... 516
Amputations and Dressings. (Williams.) .......... 268
Appendicitis, with Report of Cases. (McRae.) .......... 193
Aseptic and Antiseptic Treatment of Wounds. (Crawford.) .......... 603
Asexualization for Prevention of Crime. (Sim.) .......... 403
Beechwood Creosote in the Treatment of Phthisis. (Cox.) .......... 14
Chronic Otorrhea—Its Significance and Treatment. (Roy.) .......... 287
Conservatism in Abdominal and Pelvic Surgery. (McRae.) .......... 395
Conservatism in Abdominal and Pelvic Surgery. (Olmsted.) .......... 392
Duty of the Physician to His Patient. (Elliott.) .......... 129
Early Operation in Appendicitis. (Gaston.) .......... 587
Epsom Salts for the Pain in Burns. (Howard.) .......... 13
Essential Elements of Ideal Surgery. (Wilson.) .......... 7
Extra-Uterine Superfetation. (Kime.) .......... 399
Fatal Case of Vomiting in Pregnancy. (Nolen.) .......... 542
Granular Endometritis. Menorrhagia from Fungous Endometritis. Dysmenorrhea Due to Anteflexion. (Goelet.) .......... 156
Higher Medical Education in the South. (Elkin.) .......... 531
History of Medicine and Surgery in Georgia. (Grandy.) ......459, 597, 705
Incontinence of Urine in Children. (Satterwhite.) .......... 716
Induction of Labor in Nephritis. (Brinton.) .......... 719
Making and Closing the Incision in Abdominal Surgery. (Holmes) ... 199
Malignant Growths of the Skin: Their Diagnosis and Treatment. (Hutchins.) .......... 205
Management of the New-Born. (Holliday.) .......... 647
Migraine. (Hagan.) .......... 656
Mixed Infection. (Hutchins.) .......... 577
Morphine Poisoning Treated with Permanganate of Potash. (Darby.) 389
Nature and Symptoms of Exophthalmic Goitre. (Joffroy.) .......... 1
Nausea. (Baird.) .......... 321
Operation for Fibro-Cystic Sarcoma, Involving the Right Inferior Maxillary Bone. (Gaston.) .......... 142
Orator's Address before the Alabama Medical Association, April 17, 1894. (Blake.) .......... 212

ORIGINAL COMMUNICATIONS—*Continued*—

Parenchymatous Rupture Treatment. (Lindorme.) .... ........ ......... 664
Pathology and Treatment of Gonorrhea. (Blackford.)...... ................ 325
Plea for the Obstetric Binder. (Allen.) ......................... .......,....... 121
Psoriasis. (Gottheil.)......... ......... ............... ............... ................ 466
Scopolamine Hydrobromate. (Hobbs.)................ ............................ 401
Suggestion and Its Therapeutic Uses. (Benedict.)............................ 257
Surgical Treatment of General Peritonitis. (Allis.).... ............ ........ 723
Tetanus. (Montgomery.) .................... ........... ......... ................ 452
Therapeutics of Infancy and Childhood. (Gewinner.)........................ 337
Treatment of Gunshot Wounds. (Westmoreland.)............................ 74
Treatment of Puerperal Peritonitis. Hardon.)................. ............... 449
Treatment of Pterygia with the Galvano-Cautery. (Hobbs.)............ 284
Treatment of Syphilis by Hypodermic Injections, with Special Reference to the Salicylate of Mercury. (Amster.) .............................. 331
Treatment of Uterine Fibroids. (Goelet.)..... ... ................................ 10
Two Major Amputations. (Cooper.)........ ...................... .................. 385
Two Recent Cases of Excision of the Vermiform Appendix for Chronic Relapsing Appendicitis in the Interval. (Morton.)......... ........ 71
Uterine Cancer. (West.)......... ........................ ......... ... .................. 580
Vaginal Hysterectomy for Malignant Disease of the Cervix and Uterine Myoma. (McMurtry.)...... ................... ..... ..... .................... 63

SOCIETY REPORTS—

Atlanta Society of Medicine.................................... .........21, 223, 407
Atlanta Obstetrical Society......... .............................. .... ................... 474
Augusta Society of Medicine......................................................470, 614, 667
Clinical Society of Maryland... ......................................................16, 346 728
Georgia State Medical Association................................ ....................... 160
Macon Medical Society.............. ............. ........... ..... .................296, 344
Philadelphia Academy of Surgery.... ... ............................................... 80
Tri-State Medical Society, Alabama, Georgia, and Tennessee............ 544

CORRESPONDENCE—

A Few Surgical Observations..... ................................................... 349
Castration for Hypertrophy of Prostate Gland.................................. 733
Craig Colony for Epileptics.. ... ..................................................... ........ 674
Diphtheria and Antitoxin... .......................................... ... ................... 731
New York Letter........ .................... ........ ......................26, 87, 234, 477
Psoas Abscess.................................................................. ................ 673
Watermelon Seed in Windpipe...................... ................................ 414

EDITORIALS—

Atlanta Society of Medicine ................. ........................................ 681
Castration of Women......................................................... ........ 33
College Commencements............ ................................................. 101
Contract Service by Railway Surgeons......... ..................................... 738
Dr. Wm. Thos. Coggin.................................. ................................ 356

EDITORIALS—*Continued*—

Foot-Ball Casualties.......... 242
Higher Medical Education .......... 557
Legislation for the Prevention of Blindness.......... 484
Legislative Control of Medical Practice.......... 351
Medical Legislation in Ohio.......... 36
Medicine as She Is Taught.......... 354
More about the Serum Therapy.......... 679
Oliver Wendell Holmes.......... 559
One Board or Three?.......... 419
One Way to Fame; or, Dr. Morton and His Letheon.......... 741
Proposed Bill for Three Boards of Examiners.......... 481
Serum Therapy for Diphtheria.......... 620
Smallpox in Atlanta.......... 99
Some Homeopathic Therapeutics .......... 239
Some Misdirected Letters.......... 172
The Amick Quack Co.......... 240
The Atlanta Obstetrical Society.......... 357
The First American Symphyseotomy .......... 103
The Growth of Medical Journalism.......... 300
The Medical Association..........99, 120
The Medical Bill.......... 623
The Medical Boards.......... 737
The Medical Law.......... 676
The Meeting of the American Medical Association.......... 299
Treatment of Epilepsy, Chorea, etc... .......... 416
Visit of the Medical Editors... .......... 37
Wants to Study Medicine.......... 421

SELECTIONS AND ABSTRACTS—

Eye, Ear, Nose, and Throat..........489, 561, 744
General Medicine..........56, 115, 179, 367, 422, 564, 687
Obstetrics and Gynecology..........49, 174, 303, 431, 630, 693
Practical Chemistry and Pharmacy..........40, 111, 244, 310, 375, 748
Skin Diseases and Syphilis..........54, 109, 247, 307, 435, 492, 682
Surgery..........43, 104, 185, 359, 441, 498, 625, 751

MEDICAL ITEMS.......... 59, 118, 190, 250, 313, 378, 444, 509, 571, 633, 698

BOOK REVIEWS..........[60, 120, 192, 253, 317, 380, 446, 573 611, 635, 700

### LIST OF CONTRIBUTORS.

Allen, Jos. Eve, M.D. .......... Augusta.
Allis, Oscar H., M. D. .......... Philadelphia.
Amster, L., M.D. .......... Atlanta.
Baird, J. B., M.D. .......... Atlanta.
Benedict, S. C., M.D. .......... Athens.
Blackford, C. M., Jr., M.D. .......... Lynchburg, Va.
Blake, W. H., M.D. .......... Lineville, Ala.
Brinton, William, M. D. .......... Baltimore.
Cooper, Hunter P., M.D. .......... Atlanta.
Cox, Wm. J., M.D. .......... Flovilla.
Crawford, Wm. B., M.D. .......... Augusta.
Darby, J. L., M.D. .......... Americus.
Delafield, Francis, M.D. .......... New York.
Elkin, Wm. S., M.D. .......... Atlanta.
Elliott, W. H., M.D. .......... Savannah.
Eve, Robert C., M.D. .......... Augusta.
Gaston, J. McF., M.D. .......... Atlanta.
Gewinner, N. G., M.D. .......... Macon.
Goelet, Augustin A., M.D. .......... New York.
Gottheil, W. S., M.D. .......... New York.
Grandy, Luther B., M.D. .......... Atlanta.
Hagan, Hugh, M.D. .......... Atlanta.
Hardon, Virgil O., M.D. .......... Atlanta.
Hobbs, Arthur G., M.D. .......... Atlanta.
Holliday, W. Z., M.D. .......... Augusta.
Holmes, J. B. S., M.D. .......... Atlanta.
Howard, N. F., M.D. .......... Dahlonega.
Hutchins, M. B., M.D. .......... Atlanta.
Joffroy, Docteur A. .......... Paris.
Kime, R. R., M.D. .......... Atlanta.
Lindorme, C. A. F., M.D. .......... Atlanta.
McMurtry, L. S., M.D. .......... Lousiville.
McRae, Floyd W., M.D. .......... Atlanta.
Montgomery, Charles J., M.D. .......... Augusta.
Morton, T. G., M.D. .......... Philadelphia.
Nolen, Wm. L., M.D. .......... Chattanooga.
Olmsted, J. C., M.D. .......... Atlanta.
Page, R. C. M., M.D. .......... New York.
Roy, Dunbar, M.D. .......... Atlanta.
Satterwhite, T. P., M. D. .......... Louisville.
Sim, F. L., M.D. .......... Memphis, Tenn.
West, Geo. R., M.D. .......... Chattanooga, Tenn.
Westmoreland, W. F., M.D. .......... Atlanta.
Williams, H. J., M.D. .......... Macon.
Wilson, B. F., A.M., M.D. .......... Slater, Mo.

